# Strain Measurements within Fiber Boards. Part I: Inhomogeneous Strain Distribution within Medium Density Fiberboards (MDF) Loaded Perpendicularly to the Plane of the Board

**DOI:** 10.3390/ma5061115

**Published:** 2012-06-19

**Authors:** Jörn Rathke, Gerhard Sinn, Johannes Konnerth, Ulrich Müller

**Affiliations:** 1 Wood K plus—Competence Centre for Wood Composites and Wood Chemistry, Altenberger Straße 69, 4040 Linz, Austria; E-Mail: ulrich.mueller@kplus-wood.at; 2 Department of Material Sciences and Process Engineering, Institute of Physics and Material Sciences, BOKU—University of Natural Resources and Life Sciences, Peter Jordan Straße 82, 1190 Vienna, Austria; E-Mail: gerhard.sinn@boku.ac.at; 3 Department of Material Sciences and Process Engineering, Institute of Wood Science and Technology, BOKU—University of Natural Resources and Life Sciences, Konrad Lorenzstraße 24, 3430 Tulln an der Donau, Austria; E-Mail: johannes.konnerth@boku.ac.at

**Keywords:** electronic laser speckle interferometry, internal bond strength, medium density fiber board, strain distribution

## Abstract

Internal bond strength testing is a widely used approach for testing quality traits of wood based panels. Generally, failure of internal bond specimens is due to adhesion and/or wood failure in the specimen. It has been reported that a composite product with a large variation in the vertical density profile fails in the center part of the board which is either the middle of the core layer or the transition zone between core layer and face layer. The density in the failure zone is typically 50% lower than the maximum density in the face layers. The aim of this study was to analyze the strain distribution in a specimen under tension perpendicular to the panel plane. The results showed that a high variety of strain magnitude occurred in the specimen. The strain is either aligned with the tension direction or a tension zone is built in one of the edge zones leading to failure. Vector graphics of the specimen show the problematic test setup of internal bond strength measurement. Strain spots in the edges lead to the assumption of an uneven stress distribution due to the momentum which results from non-perfect alignment or irregularities in the test setup.

## 1. Introduction

Wood is a highly heterogeneous material with randomly distributed weak zones which act as sources of failure. To overcome this drawback, wood can be fragmented into strands, particles, as well as fibers which are finally reassembled again by means of adhesives, pressure and heat to gain homogeneous wood based panels with customized properties. Besides the orientation and the size of the particles, the entire production process, as well as the raw material used, have a big impact on the board properties. In the medium density or high density fiberboard (MDF/HDF) production, several coupled physical and chemical phenomena influence the entire production process and the final product. Numerous investigations have been performed to describe the correlation between the mechanical properties of fiberboards with adhesive application [1,2,3,4], matt forming [5], pressing [6,7,8], heat transfer [6,8] and adhesive curing [9].

The standard mechanical characterization techniques for wood based panels according to European standards include bending (EN 310), and internal bond (EN 319) [10] tests. Panel producers use these methods for quality assurance of the whole production chain. Thereby, the internal bond (IB) testing procedure provides information about the tension strength perpendicular to the surface layers. Hereby sample failure predominantly occurs in the middle layer. IB strength depends on many process related factors, such as pressure, temperature, raw material, adhesive content and adhesive type but also the material density. The correlation of the internal bond strength with processing parameters has widely been investigated. Significantly reduced IB values were determined for high moisture content [11] and low resin content [12], which predestines this test method as a quality control (QC) tool in the wood panel industry.

The internal bond strength of wood composites is defined as the ultimate failure stress of a wood composite panel under tensile load perpendicular to the board plane [13]. The basic principle of measuring the IB can be explained as follows: specimens (cross section 50 × 50 mm) are adhesively bonded to braces and tested in tension. However, the testing procedure and especially the adhesive bonding of the specimen to the load blocks have a high number of effects which can bias the IB values to a large degree. Overlap of the adhesive at the edges, incomplete bonding of the specimen to the braces, varying bond line thickness and inhomogeneous application of stresses from the braces to the specimen are some examples which induce stress concentrations within the specimen, which in turn affect the IB value measured.

The aim of IB testing is determining the quality traits in the specimens’ core layer. Thus, the position of the failure should happen in the center of the core layer which is thought to represents the weakest zone [14]. In contrast, Schulte and Frühwald [15] described a variation of failure positions in a range of 25%–75% of the panel thickness, independent of the panel thickness. Here, three average failure lines at the positions 35%, 50% and 65% of the maximum density were determined. These findings correspond to the center of the core layer and the transition zones from core to face layer. One explanation for eccentric specimen failure, given in Schulte and Frühwald [16], is the pressing process. At the beginning, the pressing process leads to plasticization and adhesive curing in the face layer, while the middle layer takes a much longer time for the curing process. In later pressing phases, responsible for the densification of the core layer, the transition zone between core and face layer has to change its structure, thereby generating a weak zone. However, no correlation of the density profile and the failure position could be found [16]. Therefore, the internal bond strength and the failure position clearly depend on additional other parameters than the density profile alone.

According to Dai *et al.* [13], the failure of IB specimen depends on the stress distribution and stress allocation in the test piece. After the failure of one of the specimen sections, others will follow as the applied stress continues to increase. This leads to further de-bonding and finally to specimen failure. In general, tension specimens have to be perfectly aligned in the load axis to be exposed to an even stress distribution. Non-perfect alignment of the specimen in the direction of the load axis causes a momentum and therefore an inhomogeneous stress distribution within the specimen. This leads to a different failure mode than expected for specimen with an evenly distributed stress field.

The aim of this study is to analyze the displacement field and the strain distribution by means of electronic laser speckle interferometry (ESPI) in medium density fiberboards while testing internal bond characteristics. The density profile analysis allows drawing conclusions regarding the failure position and the density effects. Depending on specimen positioning and preparation, a variety of stress centers across the core layer can be found.

## 2. Experimental Section

### 2.1. Internal Bond Strength Measurement

The IB specimens (50 mm × 50 mm) were cut from industrially produced 38 mm thick medium density fiber boards. Before testing, the IB specimens were bonded to aluminum braces using a fast curing cyano-acrylate adhesive (Loctite 431, Henkel Co., Düsseldorf, Germany).

The determination of the internal bond strength was performed on a Zwick/Roell Z020 universal testing machine in accordance with EN 319 (1993). The specimens were tested until ultimate failure with a continuous crosshead speed of 0.5 mm/min. The internal bond strength $f_{t}$ was calculated according to Equation (1) by dividing the maximum load $F_{\max}$ by the cross section a⋅b of the specimen.

(1) $$f_{t}=\frac{F_{\max}}{a\cdot b}$$

### 2.2. Density Profile Measurement

The density profiles of the three specimens, which were additionally tested by means of electronic laser speckle pattern interferometry (ESPI), were determined using X-ray equipment (GreCon DAX-5000, GreCon, Alfeld-Hannover, Germany). The measurement system determines the X-ray attenuation by means of a line scan every 0.03 mm along the specimen thickness. The accuracy of the device is at a failure rate of ± 1%. From the data the following characteristic features were calculated: mean density, minimum density, maximum density in the surface layers and average density in an area of ± 1 mm around the failure section.

### 2.3. Speckle Measurement

**Figure 1:**
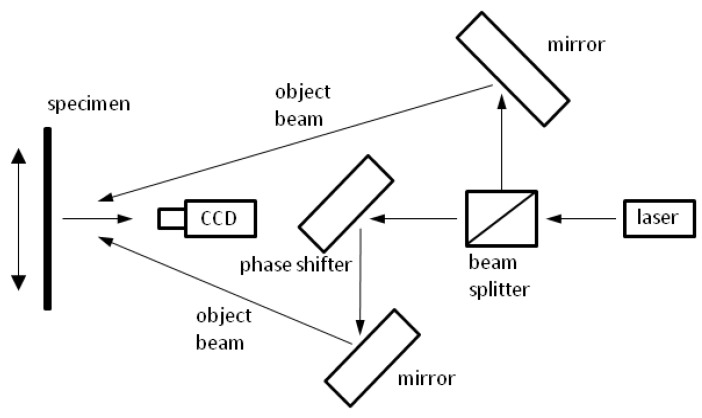
Schematic drawing of the Michelson interferometer and electronic laser speckle interferometry (ESPI) optics set-up for in-plane measurements.

Following the density profile measurement the three specimens were tested by means of electronic laser speckle pattern interferometry (Figure 1). As the field of view covered only one of the four sides of the IB specimen, deformations could only be measured in one plane.

In the EPSI measurements, deformations on the sample surface cause a change in phase difference and result in a changed speckle pattern. For the calculation, the new image pattern is subtracted from the previous image and results in an image with typical fringe pattern [17,18]. Thereby, the points along the fringes correspond to the lines of constant displacement in direction of the sensitivity vector, predetermined by the optical configuration. For in-plane measurements, the recorded intensity *l_x_*_1,*x*2_ can be written as:

(2) $$l_{\left(x1,x2\right)}=l_{R(x1,x2)}-l_{D(x1,x2)}=2\sqrt{l_{r(x1,x2)}l_{o(x1,x2)}}\cos\Delta \Phi_{\left(x1,x2\right)}$$

where $l_{R(x1,x2)}$ and $l_{D(x1,x2)}$ are the intensity of the speckled images before and after the deformation, respectively; the intensity of the reference and the object beam are described by $l_{r(x1,x2)}$ and $l_{o(x1,x2)}$, respectively; $\cos\Delta \Phi_{\left(x1,x2\right)}$ is the phase difference due to the displacement of the sample surface.

In order to analyze the stress distribution on the surface of internal bond specimens (*i.e.*, MDF), internal bond tests corresponding to EN 319 were performed on a Zwick/Roell Z020 universal testing machine. The internal bond specimens were clamped between two gimbal-mounted specimen holders.

**Figure 2:**
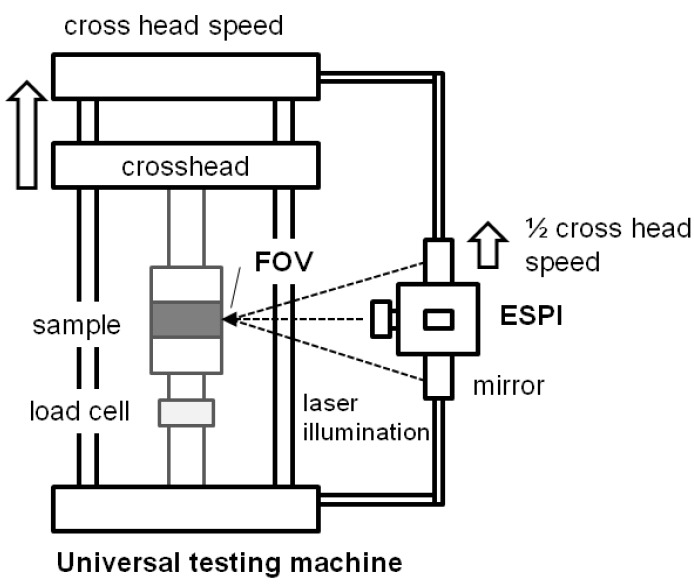
Test setup for internal bond specimen analysis in a testing machine with ESPI test setup.

A Dantec Ettemeyer Q300 (Dantec Ettemeyer, Ulm, Germany) ESPI system was mounted on the testing machine (see Figure 2). The high sensitivity of the system requires a constant control of the field of view (FOV) while the experiment is performed. A pulley system between the ESPI and the crosshead of the universal testing machine ensured that the ESPI system was moved at half the crosshead speed. This ensured a constant positioning of the ESPI system in the middle section of the specimen.

The optical axis of the ESPI system underwent the same movements during the testing procedure as the center part of the specimen. The working distance between the optical system and the specimen surface was 345 mm; a total area of 44.6 mm × 38.9 mm (FOV) was observed.

For the analysis of the total sample deformation during the whole mechanical experiment, several ESPI images were taken. In the experiment, the crosshead was moved with a speed of 0.1 mm min^−1^. Pictures were taken after three additional fringes appeared which yielded approximately 15 pictures per specimen. The test was performed until fracture occurred. After each of the steps, the crosshead was stopped for approximately 4 seconds and allows generating the ESPI image in all three directions (x_1_, x_2_, x_3_). To ensure stable conditions for the ESPI measurements (no lateral movements and no vibration), a pre-force of 20 N was applied to the specimen before the ESPI measurements began. The stack of ESPI images was stored on a computer and used for the deformation measurements after the experiment. The post-processing of the speckle images was performed with the ISTRA software. For a detailed calculation of the strain distribution and to observe strain concentrations, a smaller field of view was investigated and analyzed. The total sample deformation was calculated by summing up all deformation increments before failure occurred.

## 3. Results and Discussion

### 3.1. Internal Bond Testing

Internal bond tests were performed analyzing ten MDF specimens with a thickness of 38 mm and an average density ranging from 708.3 kg/m^3^ to 714.6 kg/m^3^. Conventional testing revealed internal bond strength values of 0.51 ± 0.19 MPa with a corresponding coefficient of variation of 37.2%. According to EN 622-5 2006 the internal bond strength of MDF has to meet 5%—fractile values of 0.50 MPa for internal usage. The tested IB specimens were found to meet these requirements according to the standard.

### 3.2. Density Profile Measurement

Density profile measurements were carried out on three specimens, which were also tested regarding to their internal bond strength and analyzed by means of ESPI. The density profile measurements showed a maximum density of 1130.1 kg/m^3^ (S1), 1138.9 kg/m^3^ (S2) and 1125.2 kg/m^3^ (S3), while minimum density ranged from 561.2 kg/m^3^ to 563.7 kg/m^3^. Additionally, density was measured at the level where failure occurred (failure density) (see Table 1).

**Table 1:** Density values and failure position related density of tested medium density fiberboard (MDF) specimens.

Specimen	Mean density (kg/m³)	Minimum Density (kg/m³)	Maximum Density (kg/m³)	Failure Density (kg/m³)
1	708.7	563.7	1130.1	590.2 (26.3 mm)
2	714.9	563.5	1138.9	589.7 (22.6 mm)
3	708.2	561.2	1125.2	587.5 (26.7 mm)

The minimum density measured is approximately 49% lower than the maximum density. A comparison of maximum density and failure density shows that failure of the three analyzed specimens occurred at a density of approximately 52% of the maximum values. As mentioned above, Schulte und Frühwald [15] found that the specimen failure density of MDF, if tested by means of internal bond strength, is highest at failure lines of 35%, 50% and 65% of the maximum density and within a failure position range of 25% to 75% of the board thickness. In our case the failure position is in a range of 37% to 64%, which was caused by the pronounced density profile.

**Figure 3:**
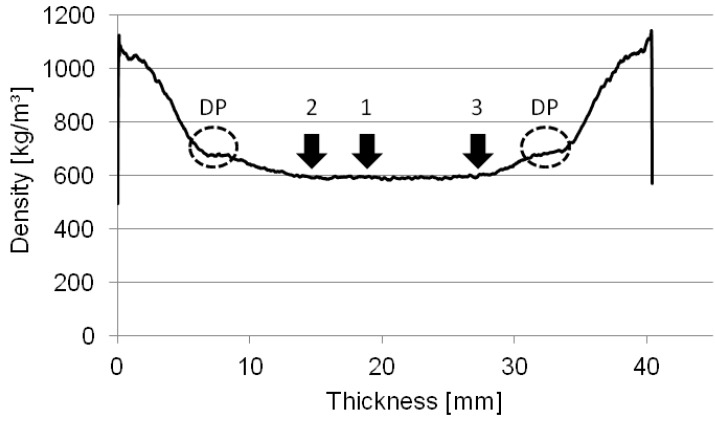
Representative density profile and density plateau (DP) of internal bond specimens with a thickness of 38 mm; corresponding failure zones of Specimens 1–3, which were analyzed by means of ESPI.

Figure 3 shows the density profiles of the three specimens tested. In the density profile the zone of re-densification is visible as a density plateau (DP) between the maximum and the minimum density. Anyhow, the analysis of the failure positions shows two characteristic regions of failure. The classic region of failure can be found in the middle of the core layer of the board (Figure 3, Specimen 1), while the second region of failure is found in the transition zone between the core layer and the inner part of the re-densification zone (Positions 2 and 3 in Figure 3).

The weak transition zone results from the pressing process. During the process, the resin hardening first takes place in the face layer. The hardening in the core layer takes much longer than in the face layer, as the steam blow and the temperature transition from the outer zones to the inner part of the board takes a certain time. In general, the pressure increases with the duration of the pressing process, which leads to a re-densification of the fiber mass. This densification process leads to a failure zone in the transition area between core layer and face layer, as, in this section, some parts of the resin are already cured while others are not. The specimen failures at the Positions 2 and 3, shown in Figure 3, can be traced back to the described production characteristic.

### 3.3. Speckle Measurement

**Figure 4:**
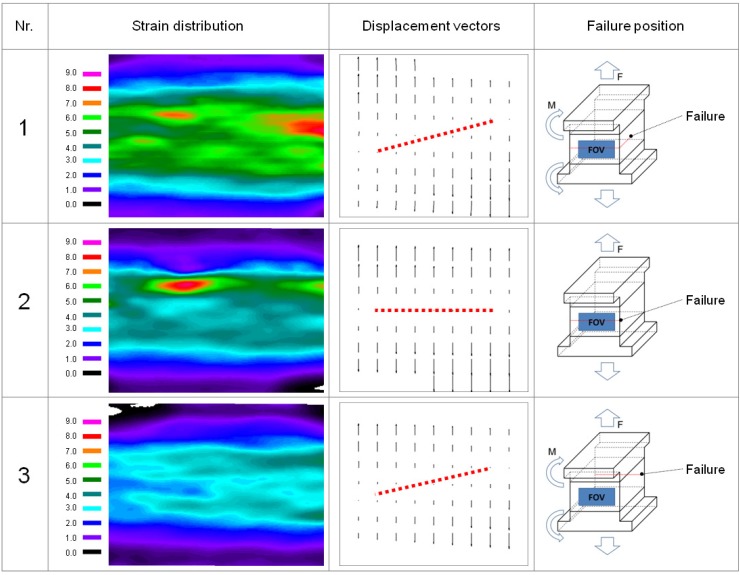
(**left**) In-plane strain distribution $\varepsilon_{yy}$ (μm/mm) (**middle**) displacement vectors of Specimens 1, 2, 3; (**right**) Specimens 1, 2, 3 with field of view (FOV), failure zone and momentum (M).

In the following section, the results of the experiments performed by means of ESPI are presented and discussed. The specimens tested are the same as the specimens shown in the density profile measurement section. Typical in-plane strain distribution maps of the internal bond specimens’ core layer sections are presented in Figure 4.

A very good distinction between the core layer and the face layer is given in the strain distribution *ε_yy_* maps of all three specimens. Specimen 1 shows the most homogeneous distribution of strain, not only in terms of the strain map, but also especially in the horizontal strain profile. The highest deformations occur in Specimen 1 in the core layer and the transition zone between the core layer and the face layer, which can be seen the strain distribution map. Specimen 2 shows, in contrast to specimen 1, the highest rate of strain in the transition zone between core layer and face layer. The measured strain is non-homogenous and focused in small regions. These strain spots are aligned in the upper part of the core layer. Specimen 3 was analyzed from a point which was found to be the opposite side of the failure zone as shown with the failure line in the right column of Figure 4. The strain distribution map shows only a small amount of strain, while in the rest of the specimen, little or negative strain was measured which is equivalent to a necking of the specimen and is probably due to vertical bending. For Sample 1, necking (−0.44 µm/mm) can be observed in the middle section of the specimen. In contrast, Specimen 2 shows no necking, but zones of high deformation in tension direction. A maximum deformation of 9.7 µm/mm was found in the center part of the specimen. While Specimens 1 and 2 revealed a fracture zone in the FOV, the fracture zone of Specimen 3 was on the backside of the specimen. This explains why, in this case, only indefinable deformations can be found. The necking was measured using the line measurement of the ISTRA software package (Dantec Ettemeyer Co., Ulm, Germany).

In the middle part of Figure 4 vector graphs of the in-plane displacement are presented. The Specimens 1 and 3 show a diagonal processing zero deformation line. This indicates a moment (M) in the test setup. As all specimens were diligently prepared for the testing and high attention was given to an accurate specimen alignment, the internal bond strength test setup is shown to react to irregularities in the test setup which cannot be influenced by conventional test setups. In contrast to Specimens 1 and 3, Specimen 2 reveals displacement vectors with a perfect alignment in tension direction.

## 4. Conclusions

This study demonstrated the applicability of ESPI to study the strain distribution of internal bond specimens. The tested reference specimens were in the range of the required internal bond strength according to EN 319. The density profile tests showed a symmetric and homogeneous density profile. The cracks in the three specimens, which were analyzed by means of ESPI, appeared in the core layer or in the transition zone from core layer to face layer. Comparing the crack position with the deformation vectors, it can be shown that, if the specimen orientation is not perfectly in the direction of load, the specimen will not necessarily fail in the middle of the core layer. Additionally, the analysis of the strain shows a highly inhomogeneous distribution. This reflects the weakness of the internal bond strength testing system: the uneven stress distribution results in imprecise data, regardless of the amount of attention paid to specimen preparation and arrangement in the test set-up.
